# Observations and Recommendations Toward Increasing Age-Inclusivity

**DOI:** 10.1093/geroni/igab046.044

**Published:** 2021-12-17

**Authors:** Nina Silverstein

**Affiliations:** University of Massachusetts Boston, Boston, Massachusetts, United States

## Abstract

Some areas of current campus practice better align with AFU principles than others. It may be that age-friendly practices already implemented by campus administrators are either not sufficiently publicized or that the campus constituents are not aware that these accommodations are in place. Specifically, staff members in these institutions appear to have markedly different perceptions of an institutions’ overall age-friendliness than did students and faculty. Lower ratings of age-friendliness by staff suggest that the experiences of ageism could contribute to negative outcomes such as stress and burnout. In line with Sustainable Development Goals (SDGs) in the evaluation of campus sustainability efforts, aging should be addressed along with other elements of diversity, equity, and inclusion. There is also a need to support faculty development for designing courses and materials for age-diverse learners. Finally, campuses might organize age-inclusivity task forces and regularly reassess their age-friendly progress.

